# Association of Melatonin Administration in Pregnant Ewes with Growth, Redox Status and Immunity of Their Offspring

**DOI:** 10.3390/ani11113161

**Published:** 2021-11-05

**Authors:** Efterpi Bouroutzika, Maria Giovanna Ciliberti, Mariangela Caroprese, Ekaterini Theodosiadou, Serafeim Papadopoulos, Sotiria Makri, Zoi-Vasiliki Skaperda, Georgios Kotsadam, Marios-Lazaros Michailidis, George Valiakos, Stella Chadio, Dimitris Kouretas, Irene Valasi

**Affiliations:** 1 Faculty of Veterinary Science, University of Thessaly, 43100 Karditsa, Greece; bouroutz@uth.gr (E.B.); etheodosiadou@uth.gr (E.T.); gkotsadam@uth.gr (G.K.); mamichailidis@uth.gr (M.-L.M.); georgevaliakos@uth.gr (G.V.); evalasi@uth.gr (I.V.); 2 Department of Agriculture, Food, Natural Resources, and Engineering (DAFNE), University of Foggia, 71122 Foggia, Italy; mariangela.caroprese@unifg.it; 3 Department of Ichthyology and Aquatic Environment, University of Thessaly, 38446 Volos, Greece; serpapad@uth.gr; 4 Department of Biochemistry and Biotechnology, University of Thessaly, 41500 Larissa, Greece; sotirina_m@hotmail.com (S.M.); zoskaper@bio.uth.gr (Z.-V.S.); dkouret@uth.gr (D.K.); 5 Department of Animal Science, Agricultural University of Athens, 11855 Athens, Greece; shad@aua.gr

**Keywords:** melatonin, antioxidant biomarkers, cytokines, IgG, lambs, prenatal stress

## Abstract

**Simple Summary:**

Melatonin is a known antioxidant and anti-inflammatory regime, while in sheep it is broadly used to accelerate the onset of the breeding season. Our recent study showed that melatonin administration during pregnancy in heat-stressed ewes improved fertility rate and number of lambs born per ewe, the redox status of the maternal organism and the produced milk quantity until weaning. In this study, we present the impact of melatonin administration in stressed ewes during pregnancy considering: (a) humoral response of both maternal organism and offspring during the first two days after parturition, (b) chemical composition and antioxidant parameters of colostrum and milk until weaning and (c) redox status of the offspring until weaning. The results indicated that melatonin improved the redox status of the offspring and the quality of colostrum. Moreover, melatonin could be administered as immune-modulatory regime, apart from antioxidant, in prenatally stressed offspring in order to cope with the crucial first days of their life, as the humoral response results suggested.

**Abstract:**

In this study, the effects of melatonin treatment on growth, redox status and immunity in prenatally stressed newborn lambs were evaluated. Thirty-seven newborn lambs were allocated into two groups (melatonin-MEL and control-CON), based on whether their mothers were treated with melatonin implants or not, respectively. All pregnant ewes were exposed to heat stress. The body weight of lambs was recorded at birth (L0), and then on days 15 (L15) and 40 (L40). Redox biomarkers [total antioxidant capacity (TAC), glutathione (GSH), thiobarbituric acid reactive substances (TBARS)] were assayed in blood samples collected from lambs on days L0, L1, L2, L5, L10 and L40. Chemical analysis and antioxidant capacity were evaluated in colostrum and milk samples collected at the same time points with blood samples. Cytokines (IL-1β, IL-6, IL-10, IFN-γ) and immunoglobulin (IgG) were assayed in blood and colostrum samples collected from ewes on days L0 and L1, and in lambs’ blood on days L0, L1 and L2. The results revealed that body weight gain of newborn lambs did not differ between the two groups (*p* > 0.05). Better redox status was found in MEL lambs until L2, as well as higher antioxidant capacity in the colostrum of MEL ewes compared to CON ones on day L0 (*p* < 0.05). In MEL ewes’ colostrum, higher protein content was measured on day L0 and higher fat content on L1 compared to CON group (*p* < 0.05). The highest level of IL-6 was found in MEL ewes on L1, with a concomitant increase of IL-10 level in MEL lambs in comparison to CON lambs on L2. Moreover, CON colostrum resulted in a higher level of IL-10 within time, coupled with an increased level of IgG found in lambs’ plasma on L2 (*p* = 0.04). This study indicated that melatonin could be administered as antioxidant and immune-modulatory regime in prenatally stressed offspring in order to cope with the crucial first days of their life. This effect of melatonin was also amplified by crosstalk between IL-6, IL-10 and IgG production, resulting in an improved quality of produced milk.

## 1. Introduction

Several studies have considered the long-term consequences of early life events on physiological processes linked to animal production. It is now well established that the phenotype of an individual can be driven by in utero environmental conditions [1]. Growth evidence supports the concept of “developmental programming” in livestock, which implies that a stimulus or insult acting during critical periods of pre- or post-natal growth and development may result in permanent programmed alterations on health and wellbeing of the offspring. A number of insults, such as maternal nutritional perturbations, heat stress and inflammation have been recognised as prominent causes of developmental programming [2,3], inducing, directly or indirectly, an inhibitory effect on the innate immunity of the offspring [1]. Perturbations to the emerging immune system might have long-term consequences in the physiology and disease risk of the offspring due to programming effects [4,5]. It has been demonstrated that dairy cows being exposed to heat stress during late gestation caused in female offspring a low blood lymphocyte proliferative response [6], thus showing that prenatal treatment influences both passive and acquired immune function in offspring [1]. However, the critical developmental stages of vulnerability of the immune system to environmental programming have not been elucidated so far and probably vary between different species [5].

Furthermore, various factors such as infections, undernutrition, stress of transfer and extreme weather conditions can cause an imbalance of reactive oxygen species (ROS) production [7]. One of the predisposing factors that could lead to elevated production of ROS is heat stress (HS) [7]. Thus, during pregnancy, an uncontrolled rise in ROS production in the maternal organism can cause more harm than profit, due to the fact that the defensive antioxidant mechanisms are not capable of reversing the ROS imbalance, which may lead to disruption of pregnancy and abortion or abnormalities to the foetuses [7]. We have recently shown that melatonin can be safely administered in heat-stressed ewes for improving the fertility rate as well as the redox status of ewes during pregnancy [8]. Moreover, melatonin may play a role in prenatally stressed offspring, possibly through the modulation of proinflammatory cytokines via redox-related mechanisms [9].

Melatonin (N-acetyl-5-methoxytryptamine) is a small indoleamine produced by the pineal gland predominantly during the dark phase of the circadian cycle [6,10]. Melatonin can be produced by immune cells and in many peripheral tissues, including male and female reproductive organs [11] and exerts pleiotropic bioactivities [12]. Indeed, it is a broad-spectrum antioxidant, free radical scavenger and an anti-inflammatory molecule [13,14,15,16] that regulates a number of physiological processes, including reproduction. As an antioxidant molecule, melatonin acts in two ways. First, it is a scavenger for ROS when it is found in low concentration in blood, and when its concentration is elevated, it induces the expression of antioxidant genes such as glutathione peroxidase (GPx), leading to high levels of GSH. This double role is crucial for embryo development since it reduces the levels of ROS in the placenta and embryo environment, allowing the foetus to develop in an environment where oxidative stress is abundant [11]. Moreover, melatonin has pleiotropic effects on different steps of inflammation, as demonstrated by the proinflammatory role at an early phase of inflammation through activation of cytokines release, such as IL-1 and tumour necrosis factor-alpha (TNF-α) [17].

The important role of melatonin in pregnancy and parturition has been well established. Owing to its lipid- and water-soluble character, maternal melatonin crosses the placenta easily and enters the fetal circulation without being modified [18], indicating that it has a direct effect on the embryo by upregulating its antioxidant capacity. This hormone plays a key role in regulation of development of foetal organs, which is critical for prevention of foetal losses and for successful adaptation of the neonate to extrauterine life [9]. Increased maternal melatonin circulating levels have been reported during pregnancy in sheep [19], rats [20] and humans [21,22,23]. The circulating melatonin acts to synchronise physiological functions, including energy metabolism. During pregnancy, the regulation of energy metabolism is crucial for the maintenance of maternal and fetal health [12], including the foetus’ capacity to produce anti-inflammatory molecules. Maternal serum melatonin levels show a diurnal rhythm that is an important signal for the foetus to entrain the light-dark rhythm of the newborns after birth [6]. Moreover, the role of melatonin in foetal programming has been discussed [9,24] and the use of melatonin has been suggested as a reprogramming agent [24].

The emerging immune system is vulnerable to insult not only during foetal life, but also through colostrum transfer. The “Lactocrine hypothesis” extends the developmental origins hypothesis beyond the intrauterine environment, through ingestion of colostrum as a conduit for the transmission of signalling molecules from mother to offspring [2]. Furthermore, prenatal stress may affect the acquisition of maternal immunoglobulins (Ig) via colostrum intake, which is prerequisite for the acquisition of passive immunity in sheep and the survival of newborns during their first hours after birth [25]. Apart from Ig, colostrum also provides antioxidants [26], defence cells (including macrophages), lipids and proteins that are sensitive to oxidation and contribute to the function of various antibacterial systems. However, colostrum itself is a source of oxidation-reduction reactions [27] and the Ig contained therein are molecules with high susceptibility to peroxidation [28]. As it was found in calves, the redox balance of the colostrum is closely associated with the IgG absorption [29]. 

Potential effects of melatonin administration on prenatally stressed offspring have not been studied in sheep. We have recently indicated the positive effects of melatonin on redox status of heat-stressed pregnant ewes. Melatonin treatment not only increased the mean number and body weight of lambs born per ewe but also led to higher milk production during the puerperium [8]. Our hypothesis was that melatonin could play a role in prenatally stressed offspring, possibly through the modulation of proinflammatory cytokines via a redox mechanism [9]. Thus, the aim of this study was to evaluate the role of melatonin administration prenatally as an antioxidant and anti-inflammatory regime in newborn lambs and in the quality of the produced colostrum/milk.

## 2. Materials and Methods

### 2.1. Experimental Overview

A total of 37 Karagkouniko breed lambs were included in the study and allocated into two groups, MEL (*n* = 18) lambs’ group and CON (*n* = 19) lambs’ one, based on whether their mothers had been treated with melatonin implants during pregnancy or not, respectively. The MEL group consisted of 5 singletons, 5 twins and 1 triplet, while the CON group consisted of 13 singletons and 3 twins. After the lambs’ birth, no intervention was made.

Pregnant ewes (*n* = 31) were exposed to heat stress for the first 100 days of pregnancy during the summer period in central Greece and were allocated into two groups, the MEL (*n* = 15) ewes’ group and the CON (*n* = 16) ewes’ one. In ewes of MEL group, melatonin implants (dose rate: 1 implant per ewe; Regulin, Ceva, Libourne, France) were inserted subcutaneously in the base of the ear 16 days before mating. The same procedure was repeated thrice every 40 days as was previously described by Bouroutzika et al. [8]. In total, each MEL ewe received 4 melatonin to ensure high levels of circulating melatonin throughout pregnancy and basal levels at parturition. After mating, ewes were fed with 300 g of ratio twice a day, 1 kg clover and 2 kg alfalfa hay per ewe, and had access to water *ad libitum*. From the 100th day of gestation, ewes with singletons were fed with 350 g of ratio twice a day, whereas ewes that bore more embryos were fed with 400 g twice a day. All ewes consumed 1.5 kg of clover and 2 kg of alfalfa hay daily and had access to water *ad libitum*. After lambing, the consumption of clover was increased to 1.8–2 kg per ewe daily.

Lambs’ body weight was recorded at birth, as well as on days 15 (L15) and 40 (L40) after birth. All lambs suckled their mothers until day 20 (L20) of life and then gradually were fed, also, with alfalfa hay and 100 g of ratio. Weaning of lambs took place 40 (L40) days after birth.

Conditions prescribed by legislation of the European Union in relation to animal experimentation procedures (Council Directive 86/809/EEC) were met during this work (licence number of experimental works: 41-bio/exp-04, approvals by Veterinary Faculty: 37/2016 and 6/2018).

### 2.2. Sampling

Blood samples were collected by jugular venipuncture (EDTA, BD Vacutainer^®^ blood collection tubes, BD, Franklin Lakes, NJ, USA) from ewes at lambing (L0), 24 (L1) and 48 (L2) h later. Moreover, blood samples were collected from lambs at birth (L0), 24 (L1) and 48 (L2) h later and then 5 (L5), 10 (L10) and 40 (L40) days after birth. Blood plasma was separated and stored at −20 °C until assayed.

Colostrum and milk samples were collected at the same time points for blood as were for colostrum on L0, L1 and L2 and for milk on L5, L10 and L40 time points. Each colostrum or milk sample was collected in 50 mL conical centrifuge tubes and they were immediately refrigerated at −20 °C.

### 2.3. Blood Antioxidant Methods

Three redox biomarkers, namely, total antioxidant capacity (TAC) as a crude index of the antioxidant potential of the samples, thiobarbituric acid reactive substances (TBARS) as a biomarker of lipid peroxidation and reduced glutathione (GSH) as the most important endogenous antioxidant molecule, were evaluated in all blood samples. All the methods were based on the protocols described by Veskoukis et al. [30,31,32,33].

In brief for the TAC assay, 20 μL of each plasma sample was mixed with 10 mM sodium phosphate buffer pH = 7.4 (480 μL) and 0.1 mM 2,2-diphenyl-1-picrylhydrazyl radical (DPPH•) solution (500 μL), and then incubated for 1 h in the dark at room temperature (RT), centrifuged (20,000× *g* for 3 min at 4 °C) and the absorbance was measured at 517 nm in a spectrophotometer (U-1900; Hitachi, Ltd., Tokyo, Japan). TAC was calculated on the basis of the mmol DPPH• reduced by the antioxidants present in the samples. For the TBARS assay, 100 μL of plasma was mixed with 35% trichloroacetic acid (TCA) (500 μL) and 200 mM Tris-HCl pH = 7.4 (500 μL), then incubated for 10 min at RT and 1 mL of 2 M Na_2_SO_4_ and 55 mM of thiobarbituric acid (TBA) were added. Following 45-min incubation at 95 °C, 1 mL of 70% TCA followed. The samples were centrifuged (15,000× *g* for 3 min at 20 °C) and the absorbance was measured at 520 nm in a spectrophotometer (U-1900; Hitachi, Ltd., Tokyo, Japan). The concentration of TBARS was calculated on the basis of the millimolar extinction coefficient of malonyldialdehyde (156 L/mmol/cm). Finally, for GSH assay 20 μL of erythrocyte lysate treated with TCA was mixed with 67 mM phosphate buffer (pH = 7.95) (660 μL) and 1 mM 5. 5-dithiobis (2 nitrobenzoic acid) (DTNB) (30 μL), then incubated for 45 min in the dark at RT and the absorbance was measured at 412 nm in a spectrophotometer (U-1900; Hitachi, Ltd., Tokyo, Japan). GSH concentration was calculated on the basis of the millimolar extinction coefficient of DTNB (13.6 L/mmol/cm). Haemoglobin concentration of erythrocyte lysate was measured using a commercially available kit.

All reagents were purchased from Sigma-Aldrich. Each assay was performed in triplicate and within 3 months of collection.

### 2.4. Colostrum and Milk Antioxidant Methods

#### ABTS•+ Radical Scavenging Assay

The 2,2’-azino-bis(3-ethylbenzothiazoline-6-sulphonic acid) (ABTS•+) radical scavenging capacity (RSC) was determined in all colostrum and milk samples collected, as previously described by Cano et al. [34] with minor modifications. Briefly, 1 mL reactions were prepared in distilled water containing ABTS•+ (1 mM), H_2_O_2_ (30 µM) and horseradish peroxidase (6 µM) in 50 mM phosphate-buffered saline (PBS; pH 7.5). The solution was vortexed, followed by incubation for 45 min at RT in the dark. Subsequently, 50 µL of colostrum or milk, at various concentrations, was added and was read at 730 nm on a Hitachi by radio beam spectrophotometer (U-1900; Hitachi, Ltd., Tokyo, Japan). In each experiment, a blank lacking the peroxidase was used, while the ABTS•+ radical solution without colostrum or milk was used as the control. The percentage RSC of colostrum or milk was calculated using the following equation: RSC (%) = [(OD_control_ − OD_sample_)/OD_control_] × 100, where OD_control_ and OD_sample_ are the optical density (OD) values of the control and the test sample, respectively. Moreover, the IC_50_ value indicating the colostrum or milk amount that caused 50% scavenging of the ABTS•+ radical was calculated. All experiments were carried out in triplicate and on at least 3 separate occasions.

### 2.5. Chemical Analysis of Colostrum and Milk

Colostrum and milk samples were assayed for proteins, fat B, lactose, solids not fat (SNF) and total solids (TS©) by using Milkoscan 133 (A/S N. Foss Electronic, Hillerod, Denmark) at the laboratory of organisation ELGO-Demetra in Larissa, Greece. The colostrum samples were diluted at range 1:10, while the milk samples at range 1:5.

### 2.6. Cytokines Assays in Blood and Colostrum Samples

Cytokines’ profile of IFN-γ, IL-10, IL-1β and IL-6 was determined in ewes’ and lambs’ plasma and in colostrum samples after an initial step for defatting by centrifugation at 4 °C for 20 min at 4000× *g*. For all colostrum samples a 1:2 dilution in PBS was made.

The ELISA for IL-10 in plasma and colostrum samples was determined according to Kwong et al. [35], with some modification as previously reported in Ciliberti et al. [36], whereas, IFN-γ evaluation was performed according to Ciliberti et al. [37]. Briefly, 96-well plates (Sterilin, Newport, UK) were coated overnight at 4 °C with 100 μL of anti-bovine IL-10 mAb and with anti-bovine IFN-γ (Serotec Ltd., Oxford, UK; 2 μg/mL) in buffer carbonate (pH 9.6). After blocking non-specific binding with PBS and Tween 20 (PBST, 0.05% Tween 20) with 3% of BSA, IL-10 (Serotec Ltd., Oxford, UK) and IFN-γ (Serotec Ltd., Oxford, UK) standards, serum or colostrum samples were added and incubated for 1 h. Biotinylated secondary anti-bovine IL-10 mAb and anti-bovine IFN-γ antibody (Serotec Ltd., Oxford, UK; 2 μg/mL in PBST/1% BSA) were added for 1 h. Subsequently, streptavidin–horseradish peroxidase (1/500 in PBS, Serotec Ltd., Oxford, UK) was added for 45 min. Finally, 3,3′, 5,5′-tetramethylbenzidine substrate solution was added to each well for 30 min, and the colorimetric reaction was stopped adding H_2_SO_4_ (2 M). All incubations were performed at RT, and after each step, the plates were washed 4 times. Plates were read at 450 nm by a spectrophotometer (Power Wave XS, Biotek, Winooski, VT, USA) and data were expressed as nanograms per millilitre for IL-10 and pg/mL for IFN-γ.

The levels of IL-6 and IL-1β in plasma samples were determined by sandwich ELISA performed in 96-well microtiter plates, according to Ciliberti et al. [36]. The sandwich was build using specific antibody against sheep IL-6 and IL-1β. All the incubations were conducted at 37 °C for 1 h, and after each step, the plates were washed 4 times. Plasma and colostrum samples were read against a standard curve obtained using scalar dilution of recombinant ovine IL-6 (Cusabio Biotech Co., Wuhan, China) and recombinant bovine IL-1β (Kingfisher Biotech Inc., St. Paul, MN, USA). Data were expressed as nanograms of IL-6 and IL-1β per millilitre. Plates were read at 450 nm by a spectrophotometer (Power Wave XS, Biotek, Winooski, VT, USA). The intra-assay CV was around 10% for IFN-γ, IL-10, IL-1β and IL-6; whereas, the average of the inter-assay calculated for low, medium and high concentration was about 7, 11 and 15% for IFN-γ, 3, 7 and 15% for IL-10, and 4, 7 and 4 for IL-1β and 2, 7, and 4% for IL-6, respectively.

### 2.7. IgG Assay in Blood and Colostrum Samples

IgG level was determined in ewes’ and lambs’ plasma samples and in colostrum samples using Sheep IgG Elisa kit (Wuhan Fine Biotech Co., Wuhan, China) following the manufacturer’s instructions. All samples were diluted in PBS and the plates were read at 450 nm by a spectrophotometer (Power Wave XS, Biotek, Winooski, VT, USA). The intra-assay CV was around 10%.

### 2.8. Statistical Analysis

All data were checked for normality; when this assumption was not satisfied, a Log transformation was performed. Statistical analysis was performed by SAS statistical program (SAS University Edition).

Statistical evaluations of the lambs’ weight, of the plasma (TAC, TBARS, GSH) and colostrum/milk (ABTS•+) redox biomarkers and of chemical components of colostrum/milk (proteins, fat B, lactose, SNF and TS©) samples collected at time-series points were compared within each group (MEL or CON) and between the two groups (MEL and CON) by using two-way ANOVA for Repeated Measures. Multiple comparisons were performed by the Tukey’s test.

Cytokines (IFN-γ, IL-10, IL-1β and IL-6) and IgG levels in plasma and colostrum/milk samples collected at time-series points were analysed by ANOVA for mixed models using the MIXED procedure with Tukey’s post-hoc correction. Treatment (melatonin), time of sampling and their interaction were used as fixed effect. Animal was a random factor nested in the treatment.

Correlations were performed for each animal in pairs for the blood redox parameters (pairs consisted of TAC-GSH, TAC-TBARS or GSH-TBARS) and within time (L0-L40). In addition, correlations were performed, in each group at the same time point, in IFN-γ or IL-10 or IL-1β or IL-6 or IgG levels between ewes’ plasma and colostrum, as well as between colostrum and lambs’ plasma. Moreover, lambs’ IFN-γ or IL-10 or IL-1β or IL-6 or IgG level from colostrum was evaluated in each group from day L0 to L1 and from L1 to L2.

The correlations were considered for every measurement throughout the study. Initially, the Pearson’s correlation coefficients *r,* were calculated in each group separately. Then, the differences of *r* coefficients between groups MEL and CON were evaluated separately for each parameter effect by using Fisher *r* to *z* transformation.

Data are shown as least square means ± standard error of the mean. Significant differences were set at *p* < 0.05 for all tests, while *p* < 0.10 was considered a tendency. All graphs were prepared using GraphPad Prism version 7.

## 3. Results

### 3.1. Lambs’ Body Weight Gain

**Figure 1:**
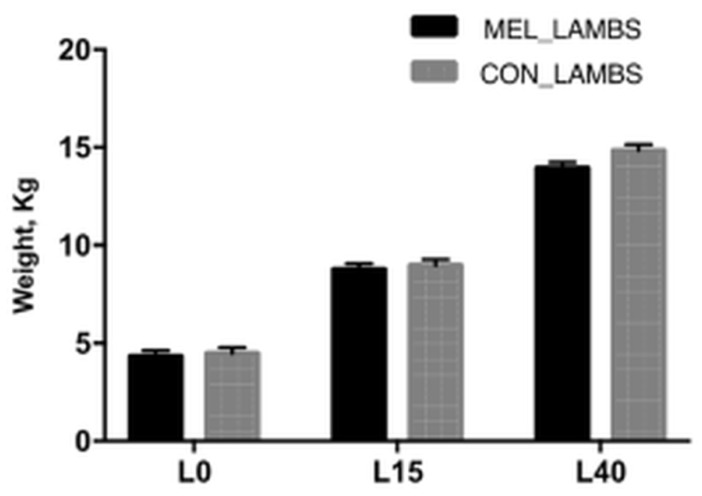
Least squares means ± SEM of body weight (Kg) of lambs, in control group (CON) and in melatonin group (MEL), at birth (L0) and on days 15 (L15) and 40 (L40) of their life, *p* > 0.05.

The mean body weight of lambs (Figure 1) did not differ between groups within time (4.03 ± 0.12 kg *versus* 3.96 ± 0.12 kg, *p* = 0.34; 8.64 ± 0.31 kg *versus* 8.95 ± 0.39 kg, *p* = 0.55 and 14.20 ± 1.94 kg *versus* 15.09 ± 2.10 kg; *p* = 0.29, on days L0, L15 and L40, respectively).

### 3.2. Antioxidant Biomarkers in Blood

**Figure 2:**
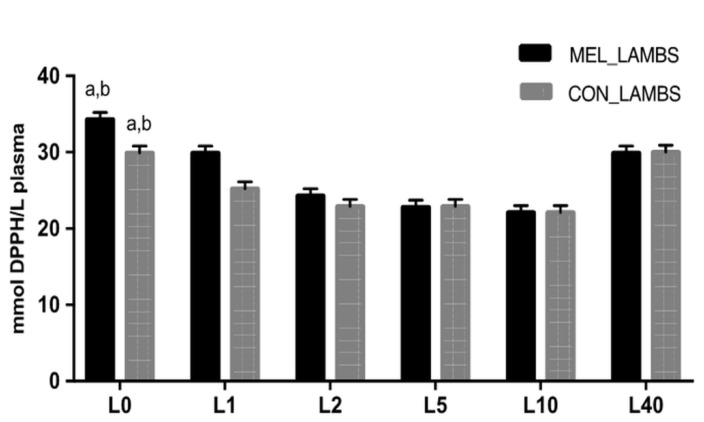
Least squares means ± SEM of total antioxidant capacity (mmol DPPH/L plasma) of lambs’ plasma, in control group (CON) and in melatonin group (MEL), at birth (L0) and on days 1 (L1), 2 (L2), 5 (L5), 10 (L10) and 40 (L40) of their life, *p* < 0.005, a: among the two groups, b: within time.

**Figure 3:**
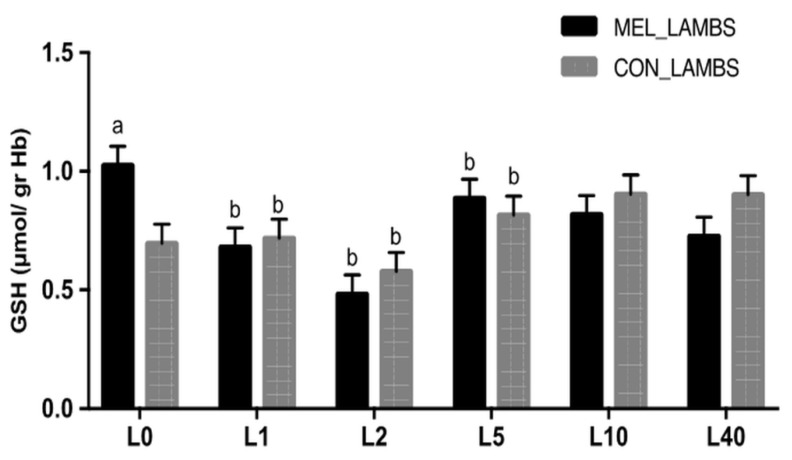
Least squares means ± SEM of glutathione levels (μmol/gr Hb) in lambs’ erythrocyte lysate, in control group (CON) and in melatonin group (MEL), at birth (L0) and on days 1 (L1), 2 (L2), 5 (L5), 10 (L10) and 40 (L40) of their life, *p* < 0.05, a: among the two groups, b: within time.

**Figure 4:**
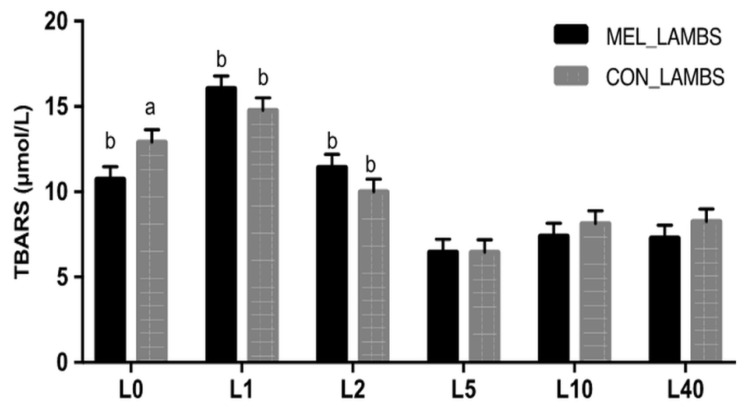
Least squares means ± SEM of lipid peroxidation (TBARS, μmol/L) in lambs’ plasma, in control group (CON) and in melatonin group (MEL), at birth (L0) and on days 1 (L1), 2 (L2), 5 (L5), 10 (L10) and 40 (L40) of their life, *p* < 0.05, a: among the two groups, b: within time.

TAC values showed significant variation within time and were different between the two groups for the first 24 h of life (L0 and L1, *p* < 0.001, Figure 2). GSH levels showed variation within time and between the two groups (Figure 3). Specifically, at birth (L0), GSH was higher in MEL lambs compared to CON ones (*p* = 0.046). In both groups, a decrease was recorded in GSH between L1 and L2 (*p* = 0.003 for MEL lambs and *p* = 0.025 for CON lambs), followed by a rise on day L5 (*p* < 0.0001 and *p* = 0.0013, respectively). The levels of lipid peroxidation (Figure 4), measured as TBARS, varied within time in both groups (*p* < 0.0005 and *p* = 0.008, respectively). TBARS levels were significantly lower at birth (L0) in MEL lambs compared to CON ones (*p* = 0.049).

Clear correlations were found in MEL group in the pair TAC-TBARS on day L0 and L1 (*r* = −0.50151, *p* = 0.0143; *r* = 0.448551, *p* = 0.0271, respectively). In CON group, correlations were found in the pair TAC-GSH on L2 and L5 (*r* = 0.215439, *p* = 0.0067; *r* = 0.133508, *p* = 0.0014, respectively), in the pair TAC-TBARS at each time point (*p* < 0.0385) apart from L2 and L10 (*p* > 0.05) and in the pair GSH-TBARS on L2 and L5 (*r* = 0.233137, *p* = 0.05; *r* = −0.08572, *p* = 0.0002, respectively). The only significant difference between the two groups was found in the pair TAC-TBARS at L0 (*z* = −1.78, *p* = 0.0375). Moreover, a difference was found in CON group in the pair GSH-TBARS on L1-L2 (*z* = 1.62, *p* = 0.05).

### 3.3. Antioxidant Biomarkers in Colostrum/Milk Samples

**Figure 5:**
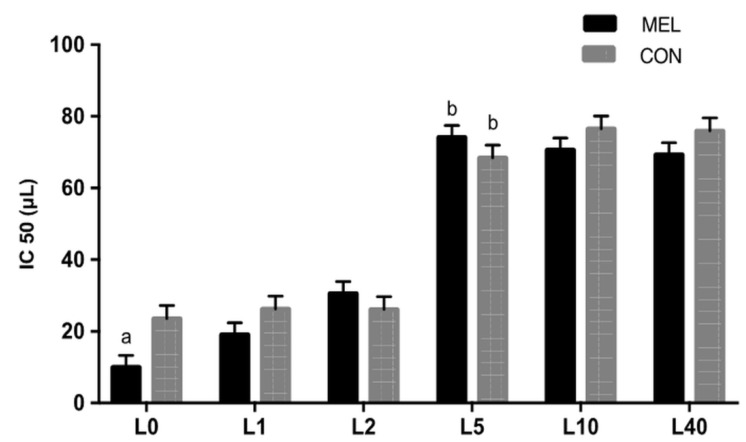
Least squares means ± SEM of ABTS•+ radical scavenging capacity (IC50, μL) in colostrum, in control group (CON) and in melatonin group (MEL), at lambing (L0) and on days 1 (L1), 2 (L2), 5 (L5), 10 (L10) and 40 (L40), *p* < 0.05, a: among the two groups, b: within time.

The ABTS•+ results (Figure 5) revealed that colostrum of MEL ewes showed greater ability of neutralisation at parturition (L0) compared to colostrum of CON ones (*p =* 0.047). Moreover, the neutralising ability decreased after day 5 (L5) in both groups (*p* < 0.0001).

### 3.4. Chemical Analysis of Colostrum and Milk Samples

**Figure 6:**
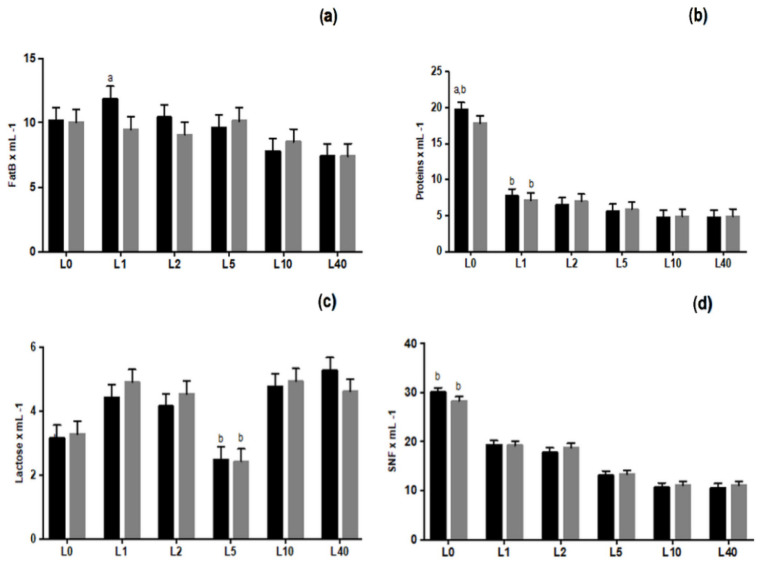

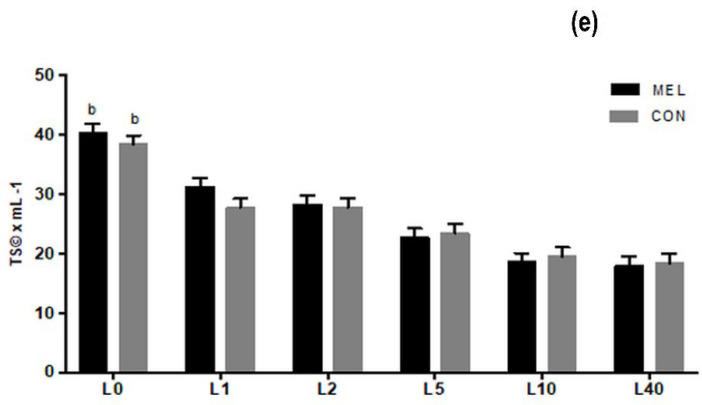
Least squares means ± SEM of chemical analysis in terms of proteins (**a**), fat B (**b**), lactose (**c**), SNF (**d**) and TS© (**e**) content in colostrum/milk, in control group (CON) and in melatonin group (MEL), at lambing (L0) and on days 1 (L1), 2 (L2), 5 (L5), 10 (L10) and 40 (L40), *p* < 0.05, a: among the two groups, b: within time.

Chemical analysis revealed that MEL-treated ewes had a higher amount of proteins (Figure 6) at lambing and higher fat content 24 h later (*p* < 0.05) compared to CON ewes. Moreover, both groups showed increased levels of SNF and TS© at lambing and decreased levels of lactose on day L5 (*p* < 0.05).

### 3.5. Cytokines Secretion in Blood of Ewes and Lambs and in Colostrum Samples

**Figure 7:**
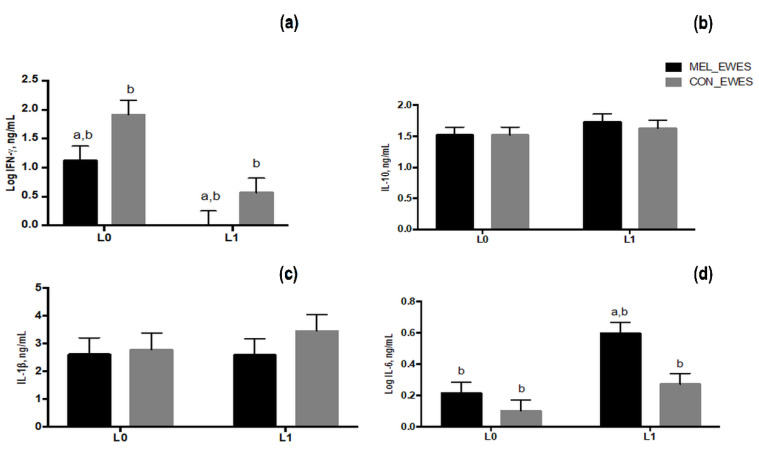
Least squares means ± SEM of levels of plasma IFN-γ (**a**), IL-10 (**b**), IL-1β (**c**) and IL-6 (**d**) at lambing (L0) and 24 h later (L1) in control ewes (CON) and in melatonin ewes (MEL), *p* < 0.05, a: among the two groups, b: within time.

IFN-γ secretion in ewes’ plasma was affected by treatment (*p* = 0.006) and time of sampling (*p* < 0.0001), registering on average a lower concentration in MEL than in CON ewes; a decreased level on day L1 as compared to L0 was found in both experimental groups (Figure 7a). No significant differences emerged for both IL-10 (Figure 7b) and IL-1β secretion (Figure 7c). Whereas, the level of IL-6 was significantly affected by treatment (*p* = 0.006) and time of sampling (*p* = 0.0008). In particular, the MEL ewes registered the highest IL-6 level on day L1 in comparison with MEL ewes on L0 and CON ewes both on L0 and L1 (Figure 7d).

**Figure 8:**
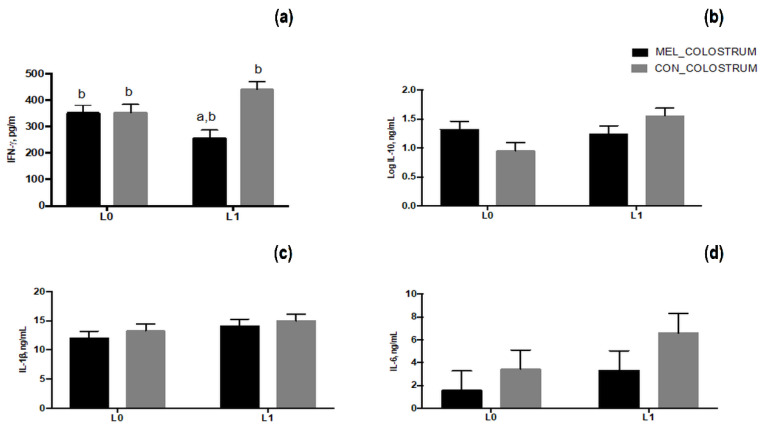
Least squares means ± SEM of levels of colostrum IFN-γ (**a**), IL-10, (**b**), IL-1β, (**c**) and IL-6 (**d**) at lambing (L0) and 24 h later (L1) in control ewes (CON) and in melatonin ewes (MEL), *p* < 0.05, a: among the two groups, b: within time.

The level of cytokines in colostrum samples is presented in Figure 8. IFN-γ secretion in colostrum was significantly affected by treatment (*p* = 0.013) and by the interaction between treatment and time of sampling (*p* = 0.015). On average, colostrum of MEL ewes had lower level of IFN-γ than CON ewes; this reduction was significant on day L1 (Figure 8a). IL-10 showed a tendency for the interaction treatment and time of sampling (*p* = 0.06) with no significance of treatment (*p* = 0.85) and of time of sampling (*p* = 0.13). In particular, colostrum of CON ewes had increased levels of IL-10 on day L1 in comparison with L0 (Figure 8b). Both the IL-1β (Figure 8c) and IL-6 (Figure 8d) did not show any significant effect of treatment, time of sampling and their interaction.

**Figure 9:**
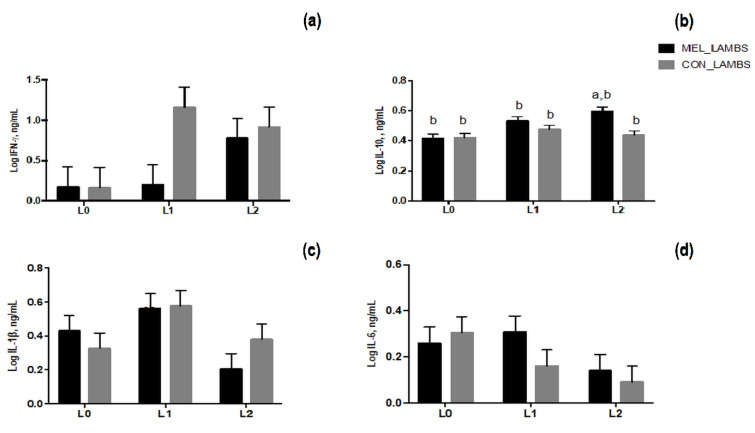
Least squares means ± SEM of levels of plasma IFN-γ (**a**), IL-10 (**b**), IL-1β (**c**) and IL-6 (**d**) secretion at birth (L0), 24 h (L1) and 48 h later (L2) in lambs born from control ewes (CON) and from melatonin ewes (MEL), *p* < 0.05, a: among the two groups, b: within time.

The IFN-γ level in lambs’ plasma was significantly affected by time of sampling (*p* = 0.0431, Figure 9a) as demonstrated by the higher level found on average on L2 than on L0. Furthermore, for IL-10 production a significant effect of treatment (*p* = 0.01), time of sampling (*p* = 0.008) and their interaction (*p* = 0.05) was registered. On average, IL-10 was lower in lambs’ plasma on L0 and increased from L1 to L2. Moreover, the MEL lambs on day L2 showed an increase of IL-10 level in comparison with CON lambs and a higher level than that found at birth in both experimental groups (Figure 9b). On the contrary, IL-1β was affected by time of sampling (*p* = 0.037), with no significant effect of treatment (*p* = 0.74) and by the interaction between treatment and time of sampling (*p* = 0.40, Figure 9c); on average, the level of IL-1β decreased from day L1 to L2. No significant effect for IL-6 secretion was registered (Figure 9d).

For IFN-γ, clear correlation was found in MEL group regarding lambs’ IFN-γ intake of colostrum during the transition from L1 to L2 (*r* = 0.888, *p* = 0.0016). In the CON group, a clear negative correlation was found between the amount of IFN-γ in the colostrum and the levels of them in lambs’ blood (*r* = −0.825, *p* = 0.0059). In the same group, a clear correlation resulted regarding lambs’ IFN-γ intake of colostrum during the transition from L0 to L1 (*r* = 0.805, *p* = 0.008). The Fischer *r* to *z* transformation revealed significant difference in the compared groups (MEL and CON) for IFN-γ levels between colostrum and lambs’ plasma on L0 (*z* = 1.64, *p* = 0.05). Furthermore, the same test showed that on L2, lambs’ ability to absorb the amount of IFN-γ, contained in colostrum of day L1, differed between the two groups (*z* = 1.86, *p* = 0.0314). Finally, the same test in CON group showed that the IFN-γ levels between colostrum and lambs’ blood differed within time (L0 and L1) (*z* = −2.04, *p* = 0.0207).

For IL-1β, none of the correlations performed were statistically significant (*p* ≥ 0.087). The Fischer *r* to *z* transformation revealed significant difference in MEL group within time regarding IL-1β concentration in colostrum on L0 and lambs’ plasma on L1 compared to colostrum on L1 and lambs’ plasma on L2, respectively (*z* = 2.16, *p* = 0.0154). Moreover, the same test showed that at L1, lambs’ ability to absorb the amount of Il-1β, contained in colostrum of day L0, differed between the two groups (*z* = 1.72, *p* = 0.0427).

For IL-10, clear correlations were found in both groups at the same time points (L0 and L1) with regard to IL-10 levels between lambs and colostrum (Mel group: *r*_L0_ = 0.664, *p* = 0.0181; *r*_L1_ = −0.607, *p* = 0.0314; CON group: *r*_L0_ = 0.762, *p* = 0.014; *r*_L1_ = 0.703, *p* = 0.026). Moreover, a clear correlation resulted in lambs’ IL-10 intake on L1 of colostrum produced on L0 in MEL group (*r* = 0.936, *p* = 0.0322). The Fisher *r* to *z* transformation revealed significant difference in the compared groups (MEL and CON) of IL-10 levels between colostrum and lambs’ plasma on L0 (*z* = −2.1, *p* = 0.0179). Furthermore, the same test showed that on L1, lambs’ ability to absorb the amount of IL-10, contained in colostrum of day L0, differed between the two groups (*z* = 2.82, *p* = 0.0024).

For IL-6, significant correlations were found in CON group at both time points (L0 and L1) with regard to IL-6 levels between ewes’ plasma and colostrum (*r*_L0_ = 0.819, *p* = 0.0065; *r*_L1_ = 0.696, *p* = 0.0413). The Fisher *r* to *z* transformation revealed significant difference in the compared groups (MEL and CON) in IL-6 levels between colostrum and lambs’ plasma on L0 (*z* = −2.52, *p* = 0.0059) and on L1 (*z* = −3.9, *p* = 0.000); and in IL-6 levels between ewes’ plasma and colostrum on L1 (*z* = −3.85, *p* = 0.0001). Moreover, the same test in MEL group showed that the IL-6 levels between ewes’ plasma and colostrum differed within time (L0 and L1) (*z* = 3.64, *p* = 0.0001).

### 3.6. IgG Levels in Blood and Colostrum Samples

**Figure 10:**
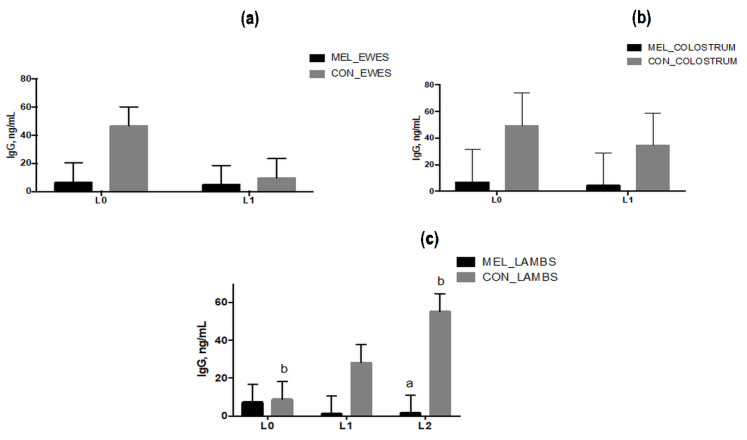
Least squares means ± SEM of levels of plasma IgG (**a**) and of colostrum IgG (**b**) at lambing (L0) and 24 h later (L1) in control ewes (CON) and in melatonin ewes (MEL) and (**c**) of plasma IgG at birth (L0), 24 h (L1) and 48 h later (L2) in lambs born from control ewes (CON) and from melatonin ewes (MEL), *p* < 0.05, a: among the two groups, b: within time.

IgG level in ewes’ plasma (Figure 10a) and in colostrum (Figure 10b) was not affected by treatment (*p* = 0.12 for ewes’ plasma and *p* = 0.18 for colostrum) and time of sampling (*p* = 0.18 for ewes’ plasma and *p* = 0.7347 for colostrum). On the contrary, in lambs’ plasma the level of IgG was significantly affected by treatment (*p* = 0.001) and the interaction between treatment and time (*p* = 0.03, Figure 10c). On average, lambs born from CON ewes registered a higher level of IgG than lambs born from MEL-treated ewes; furthermore, in lambs born from CON ewes, the level of IgG increased from day L0 to L2 (*p* = 0.04).

Regarding IgG correlations, none of them were statistically significant (*p* ≥ 0.110). The Fischer *r* to *z* transformation revealed significant differences in MEL group within time; in particular, IgG levels between ewes’ plasma and colostrum differed during the transition from L0 to L1 (*z* = 2.52, *p* = 0.0059); IgG concentration between colostrum and lambs’ plasma differed during the transition from L0 to L1 (*z* = −2.36, *p* = 0.0091). Moreover, the same test showed that on L1, lambs’ ability to absorb the amount of IgG, contained in the colostrum of day L0, differed between the two groups (*z* = −1.62, *p* = 0.0526).

## 4. Discussion

The present study aimed at the evaluation of the effect of melatonin treatment in prenatally stressed lambs on redox status and immune response, as well as on the quality of produced colostrum/milk. Our data revealed that melatonin exerted an antioxidant effect and an immunomodulatory role during the first crucial hours of newborn lambs’ life. To our knowledge this is the first study that evaluated the positive effect of maternal melatonin treatment throughout embryonic development on redox status in conjunction with immune competence of newborn lambs. Additionally, this study indicated that quality assessment of colostrum should be evaluated along with chemical composition, the IgG levels and redox status.

Melatonin can protect animals from oxidative stress, even during heat wave exposition [8,38,39]. The peculiar properties of melatonin are supported in our recent study by the increase of GSH levels in the melatonin-treated pregnant ewes followed with simultaneous decrease of TBARS almost from the beginning of pregnancy until 48 h post-partum [8]. Further evidence on the antioxidant effect of melatonin is provided by its ability to reduce lipid peroxidation, a degradative phenomenon involved in the pathogenesis of many diseases [40]. A result of great significance, in the present study, is that MEL lambs were born with better redox profiles compared to CON lambs, supporting their immune competence. Melatonin is known to promote GSH production, an endogenous antioxidant, by direct action to GPx [41], in order to reverse oxidative stress. This effect was confirmed in MEL lambs at birth, which resulted in a concomitant increase of GSH levels and lower levels of lipid peroxidation. The latter is further supported by the comparison of GSH-TBARS *r*’s in the two groups within time. Fisher *r* to *z* transformation revealed that the available GSH in CON group was as a whole offered to reduce TBARS, whereas in MEL group, modifications within time were not observed.

Maternal melatonin crosses the placenta unaltered, being an important factor for entraining circadian rhythms of foetuses. Since melatonin is also detected in milk, it contributes to the newborn’s circadian entrainment as well [42,43,44]. Melatonin is a scavenger of both oxygen and nitrogen-based reactive species, and promotes the production of endogenous antioxidants, which leads to a better adaptation of both maternal organism and embryo to heat stress conditions. The amine is a much more potent antioxidant than many traditionally used antioxidants and has two unique features. First, melatonin does not undergo redox cycling and once oxidised, cannot be regenerated to its reduced form [45]. Secondly, the antioxidant action of melatonin involves the donation of two electrons instead of one; therefore, melatonin does not become a free radical in the antioxidant process [45]. Furthermore, melatonin can be metabolised to both kynurenic acid and to N1-acetyl-N2-formyl-5-methoxykynuramine and N1-acetyl-5-methoxykynuramine [38]. These metabolites are considered very powerful antioxidants, cyclooxygenase-2 inhibitors and potentially selective anti-inflammatory agents [13,14,15]. Melatonin induces the expression of the Nrf2 gene and suppresses that of NF-κB through epigenetic processes [9,46]. In addition, melatonin acts as an anti-inflammatory agent, preventing the translocation of NF-κB to the nucleus, thus reducing the upregulation of proinflammatory cytokines [13].

Colostrum is a complex biological fluid composed of water, proteins, carbohydrates, lipids, vitamins and minerals. Colostrum is secreted by the mammary gland immediately after parturition and provides nutrition, immunity and defence, as well as growth factors to the newborn [47,48]. In the present study, the last implant was inserted on day 100 of gestation to ensure high levels of melatonin during lactogenesis-colostrogenesis. Indeed, the antioxidant effect of melatonin was clear in colostrum on L0 in MEL ewes, as well as in the TBARS levels in MEL lambs’ plasma. Given the fact that melatonin enhances the production of antioxidant peptides and their presence in the colostrum [47], it could be assumed that the higher antioxidant capacity of colostrum from MEL ewes was attributed to this effect. Moreover, the colostrum of MEL ewes showed higher fat content 24 h later compared to CON ewes. According to others, higher fat content was found in colostrum of ewes treated with melatonin in their third month of pregnancy but no alteration was found in protein content [49]. Molik et al. [50] have shown that melatonin treatment increases protein and fat content of milk, which is in agreement with our results.

It is known that birth weight *per se* is only a reflection of an insult or multiple insults to the foetus during development, indicating that birth weight is not the clearest reflection of a positive interference’s impact in developmental programming [51,52]. In the current study, no differences were observed in the birth weight between MEL lambs and CON ones, although the majority of MEL lambs were twins or triplets in contrast to CON lambs that were mainly singletons. Moreover, no difference was found in the body weight gain between the two groups until weaning, which is consistent with the results of other studies [53]. This finding may also be due to the higher milk yield measured in MEL-treated ewes than in CON, as we have previously described [8]. Perhaps melatonin treatment throughout embryonic development in MEL lambs improved their birth weight.

The immunoglobulins are certainly the most important proteins of colostrum. In ruminants, the placentation types prevent the in utero transfer of maternal immunoglobulins. For this reason, newborn ruminants rely on the ingestion and absorption of maternal immunoglobulins, especially IgG, from colostrum [47,54,55]. They are important for subsequent protection against neonatal infectious diseases and other post-partum environmental challenges [55,56], as the neonate’s immune system is not fully functional. On this basis, colostrum proteins can be divided into two major categories: (i) high abundance proteins, mainly immunoglobulins and caseins, and (ii) a wide range of low abundance proteins (acute-phase proteins, antimicrobial proteins/peptides, cytokines, growth-promoting components) (12–15). Data from the present study demonstrated the passive IgG transfer from colostrum to the lambs in both experimental groups, which is in line with the IgG level found in ewes’ plasma. Although no difference was found in IgG levels in ewes’ plasma and colostrum between the two groups, lambs born from CON ewes exhibited a higher level of IgG than lambs born from MEL ewes; whereas, the MEL lambs absorption capacity was gradually increased from birth to 48 h after birth, probably due to a better response to oxidative stress that occurred at birth. Furthermore, the increased level of IgG in CON lambs could be explained by the concomitant high level of IL-10, as demonstrated by Rousset et al. [57], in which the proliferative effect of IL-10 on B cells activated via stimulation of Ig secretions was ascertained.

On the other hand, it could be assumed that melatonin treatment prioritises the presence of antioxidant peptides in the colostrum [47] over other proteins, including IgG, as was confirmed in MEL lambs by the lower levels of IgG and better redox status. According to previous studies, a decrease in IgG was shown three days after melatonin injection in lactating cows, with a concomitant decrease in somatic cell count in milk of cows [58,59] and ewes’ milk [60]. Contrary to the latter study and to the present one, others found a higher amount of IgG but no higher amounts of proteins in colostrum just after parturition in melatonin-treated ewes that lambed males compared to females [49]. Accordingly, although different experimental designs were followed, the lower levels of IgG in MEL lambs *versus* CON ones of the current study could also be attributed to the higher milk yield of their mothers, as we have recently described [8]. Furthermore, melatonin effects on humoral immune responses could be connected to the time of melatonin administration as reported by Carrillo-Vico et al. [61]. In particular, the immuno-enhancing action of melatonin in vivo was more evident during a condition of immune depression and/or when the melatonin is administered in the late afternoon or evening. This last concept could account for the absence of clear effect of melatonin in modulating the immune system in sheep [62]. In our experimental design, the melatonin release occurred in a slow-releasing manner subcutaneously and probably this type of protocol does not imply a boosted humoral response.

Once the lamb is 24 h old, transport of IgG across the intestinal epithelium is virtually complete [63], with no further possibility of significant IgG absorption from colostrum. Fisher *r* to *z* transformation revealed that absorption capacity was reduced in MEL lambs 48 h after birth in conjunction with the better redox status found at the first days of life in MEL lambs; whereas, in CON ones, this was absent. Furthermore, the same test revealed that the amount of IgG in colostrum of MEL ewes increased within time, as well as lambs’ capacity to absorb it during the first 24 h of life (*p* = 0.0059 and *p* = 0.0091, respectively). These findings are in line with others found in calves in which the redox balance of the colostrum was closely associated with the IgG absorption [29]. Likewise, Kamada et al. [64] indicated that colostrum supplementation with selenium increased IgG absorption because selenium is a confirmed antioxidant. In our study, MEL lambs not only resulted in a better redox profile, but probably the better redox status of the colostrum on L1 contributed to attenuate the absorption capacity of the consumed colostrum.

Several studies have demonstrated a decline in immune function during the periparturient period in dairy cows; however, the causes of immunosuppression are not completely understood. It is certain that immunosuppression has been linked to endocrine changes associated with parturition, metabolic stresses associated with lactogenesis and availability of critical nutrients, including vitamin E and calcium [65,66]. Caroprese et al. [67] reported that cell-mediated and humoral immune responses and plasma IL-6 level underwent marked fluctuations in periparturient ewes. In the study of Theodorou et al. [68], two of the total three studied Greek dairy sheep breeds exhibited signs of mild immunosuppression during periparturient period. Notably, immune suppression around calving can be measured in terms of lymphocyte proliferation in vitro, antibody secretion and cytokine release [69,70]. Both cell-mediated and humoral responses are activated and regulated by proinflammatory cytokines, such as IL-6 and IL-1β. Both IL-6 and IL-1β are considered the main mediators of the secretion of other proinflammatory cytokines and of the acute phase protein synthesis by the liver [71,72].

IL-6 has a wide range of immunological functions, including stimulation of B cells and cytotoxic T cells [73]. Release and activity of IL-6 are strongly controlled and mainly occur under inflammatory conditions, being partially responsible for the increase of certain acute phase proteins [74]. Winter and Colditz [75] found elevated concentrations of IL-1β and IL-6 in ewes’ milk after experimental mammary infection with *Staphylococcus epidermidis*. The lack of consistent IL-6 trends in blood and milk could be attributed to the fact that this cytokine is likely to be quickly utilised by lymphocytes after having been secreted or inactivated by soluble receptors as described for human IL-6 [73,76]. Hence, our results highlighted the inopportunity of considering IL-6 secretion in milk as an indicator of the physiological stress connected to parturition, in contrast to IL-6 concentration in plasma, which appeared to be a suitable indicator for this purpose. Moreover, during the transition period, the level of IL-6 peaked at parturition then decreased throughout the post-partum period [67]. However, when flaxseed supplementation was offered to ewes, the levels of IL-6 measured after 14 days post-partum increased compared to CON ewes [77]. Similarly, in the present study, the level of IL-6 of MEL ewes at 24 h post-partum increased in comparison to CON ewes.

In a study conducted by Fernandez et al. [78] in healthy lambs, it was reported that the serum concentration of IL-10 increased progressively over time and the concentration peaked at day 28 [78]. Accordingly, our data demonstrated, for MEL lambs, a rise in IL-10 secretion within time. It has been demonstrated that IL-10 exerts a potent anti-inflammatory/immunosuppressive property in vitro [57], with both immunosuppressive and immunostimulatory effects on adaptive immune cell mediators via stimulating B-cell functions [79]. Furthermore, in our previous study, a positive correlation between IgG and IL-10 levels has been observed, further supporting the involvement of IL-10 in IgG synthesis in the mammary gland, as has been previously suggested [80]. This statement could help to explain the increased level of IL-10 in colostrum and the enhancement of IgG level in CON lambs 48 h after birth.

The proportion of T lymphocyte subsets changes during parturition into Th2 phenotype expression, favouring to the secretion of Th2 cytokines (IL-10, IL-4, IL-13), and during mid lactation into Th1 phenotype expression favouring the Th1 cytokines (IFN-γ and IL-12) [65,81]. In particular, the Th1 cells can activate cellular immunity and inflammatory responses, whereas the Th2 cells control humoral immunity and promote anti-inflammatory responses. The increased level of IL-10 might have a direct suppressive effect on the Th1 cell response by reducing the secretion of IFN-γ [82]. Accordingly, in the present study, the positive correlation found in IL-10 level between colostrum and lambs’ plasma could explain the low level of IFN-γ in MEL colostrum coupled with the high level of IL-10 found in MEL lambs. Moreover, the IFN-γ intake from colostrum by MEL lambs seems to increase as the time passes according to the correlation mentioned above. The latter pattern is observed for IL-10, too.

Interleukin-1 is known as an inflammatory cytokine involved in lymphocyte activation and is implicated in acute-phase response [83], host defence against bacterial and viral infections [75] and stress responses [84]. The production of detectable concentrations of IL-1β arises when there is a strong immune challenge [71,85]. Moreover, other research groups failed to detect IL-1 in milk from cows immunised with *Staphylococcus aureus* α-toxin [86]. In the present study, the absence of IL-1β concentrations in both ewes’ plasma and colostrum samples may be related to absence of acute infection caused in animals.

An interesting result occurred during the tested time period. In the MEL group, there was a tendency for increase in the produced amounts of IgG, IL1-β, IL-10 and INF-γ in colostrum or the absorbed ones by lambs, confirmed either by the correlations or “Fisher *r* to *z* transformation”. IL-6 demonstrated the opposite tendency in MEL, but in the CON group no tendency for all cytokines and IgG was found at all. This finding might support the immunomodulatory role of melatonin treatment in the offspring.

## 5. Conclusions

Early life events, including in utero environmental conditions, affect physiological processes and, as a consequence, animal production. Melatonin treatment prenatally probably assisted the newborn lambs in overcoming the oxidative stress connected to birth by supporting the antioxidant defences, reducing ROS production in lambs’ plasma and improving the free radical scavenger capacity of colostrum. Moreover, melatonin contributed to an improvement of colostrum/milk composition, in terms of protein and fat content. The cytokine profile and IgG secretion and intake further support the immunomodulatory role of melatonin. Based on our recent study [8], it could be assumed that melatonin administration throughout pregnancy in heat-stressed ewes could be a management practice not only for the pregnant females but also for their offspring to overcome the oxidative stress after their birth. These potential benefits for lowering vulnerability to insults of offspring are notable for further research focus.

## Data Availability

The data presented in this study are available on request from the corresponding author.
